# Emotional Experiences of Dementia Caregiving Transitions

**DOI:** 10.1093/geroni/igaa057.236

**Published:** 2020-12-16

**Authors:** Emily Ihara, Catherine Tompkins, William Kennedy, Rhea Vance-Cheng, Bianca Kwan, Kendall Barrett

**Affiliations:** George Mason University, Fairfax, Virginia, United States

## Abstract

Research indicates that family caregivers of individuals living with dementia are at risk for high levels of stress, depression, physical health declines, and illness. The health and well-being of family caregivers is critically important to a long-term care system that is dependent on them to continue their caregiving role. In-depth individual and focus group interviews of 16 dementia caregivers were conducted to explore the emotional experiences of caregiving stress during transitions of individuals living with dementia to a higher level of care. Data were transcribed verbatim, checked for accuracy, and analyzed by at least two members of the research team. Line-by-line coding, memo writing, and constant comparative analyses were conducted until redundancy, when no new themes were discovered. Caregivers described various levels of feeling overwhelmed and symptom progression leading to the move to a nursing facility. Social isolation featured prominently, with caregivers describing a gradual erosion of their social network and socializing opportunities because of their caregiving responsibilities and the care recipient’s deteriorating symptoms. Caregivers described feeling isolated and stigmatized. One caregiver said, “…you’re being less invited, you’re being less involved. People don’t know how to deal with you…I don’t know if they become the pariah or I become the pariah.” At the same time, maintaining social connections and having help with caregiving featured prominently in the coping mechanisms described. The health of caregivers is equally as important as the person living with dementia, and programs, interventions, and resources should be a priority for supporting families through transitions.

